# Event and Comment

**Published:** 1917-05

**Authors:** 


### DENTAL REGISTER

Vol. LXXI MAY, 1917 No. 5

### EVENT AND COMMENT

CONSERVATISM IN PRACTICE. We are printing in this issue some comments on the misinterpretation of X-ray pictures which all dentists who are relying upon this means of making their diagnoses of hidden pathologic conditions should read as they come from two of our most expert operators and students of dental disorders. It undoubtedly is our duty to avail ourselves of every facility for getting definite and positive information in regard to the conditions we are to treat, but it is always the case that overconfidence and enthusiasm with newly found facilities may lead to improper deductions and wrong procedures. There are so many chances for our picture to fail because of an improper technique in its production, or because it can not be interpreted to harmonize with our customary findings, and we have, therefore, no basis for exact judgment, all because we are so apt to misread the evidence it furnishes.

The notion some dentists seem to have that the shadow picture must be true and that it can not fail to show things as they are is so deceiving many times that we lack confidence in this means of diagnosis. Unquestionably the X-ray is one of the most valuable accessories we possess for detecting actual conditions in the apical region, and it generally supplies the much-needed evidence to enable us to decide on a definite technical procedure. We have no desire to discredit the use of the X-ray as an adjunct to practice, but we are convinced from experience that it has its limitations and is being discredited by those who use it without discretion. We recently saw a case of apparent fracture of the maxilla which seemingly gave proof of a fracture of the ramus, which other methods of diagnosis did not bear out, and which after more carefully made skiagrams were produced the fracture was disproved. This was a case which might have involved a skillful dentist in a legal suit for malpractice, but which closer scrutiny and more careful diagnosis cleared up satisfactorily.

The present agitation for more careful root canal work in connection with crown and bridgework and for the elimination of foci of infection, is leading us to take large chances with the evidence furnished by the X-ray. We have supposed that all evidences of rarefaction near the apical region indicate disease, and contra-indicate the retention of teeth in the mouth which seem to be affected, and perhaps we are justified in clearing up all such suspected areas, in spite of the fact that we know by experience that these apparently diseased areas are in no way detrimental to the patient's health. We have no doubt further experience with the X-ray will develop a better technic and that we shall develop a more accurate knowledge of the art of properly reading these pictures, and, consequently, make a more scientific application to the art of diagnosis. In the meantime we shall be wise to retain all the other approved methods of determining diseased conditions of obscure or hidden tissues.

---

### OUR GOVERNMENT IN NEED OF DENTAL HELP

---

At the outbreak of this great world's war Canada was in as great a state of unpreparedness as the United States; perhaps greater. At the call to arms volunteers came from all parts of the Dominion, but thousands of these men, willing to fight for their country, were found to be physically unfit, because of the condition of their teeth. The dentists of Canada responded nobly. Dentists all over the land offered to care for the teeth of these volunteers and thus make them fit for service. Literally tens of thousands of dental operations were performed, and it was largely through this dental aid that Canada has sent such an efficient body of men overseas.

And dentistry has received its reward in Canada. The Government, recognizing the signal services rendered by its dentists, reorganized its army dental corps, making it a separate and distinct unit, cooperating with but no longer controlled by the medical corps. Thus there are already many dentists holding high commissions; lieutenants, captains, majors and lieutenant-colonels.

And now the call has come in this country. Our Government may soon require soldiers in a hurry. The call for volunteers in time of peace has not been ardently answered, but if war comes there is no doubt that the chivalry of our young men will cause them to enlist by the thousands. Then will our Government recognize how wholly inadequate its present dental corps will be; inadequate in numbers, not in quality. Foreseeing this the National Security League has addressed the following letter to National Secretary Otto U. King.

''The suggestion has been made and we think it worthy of careful consideration, that there would be a large number of dentists in different parts of this country who would be willing to offer their services to the Government for the purpose of attending to the teeth of the men who are at present rejected on account of bad teeth. If such an arrangement could be made we think that it would then be possible for these men to be accepted for service and to begin their training without loss of time either to the Government or to themselves. This treatment would, of course, be given by the dentist under Government supervision, so that there would be no chance of the dentist being imposed upon by men not actually enlisted for service.

"We would greatly appreciate a letter from you stating whether in your opinion this plan is feasible or not. We have taken the liberty of writing to you as we consider that, as Secretary of the National Dental Association, you are in a position to sound the feeling of the great body of men composing your association."

Almost as though in response to the above the Preparedness League of American Dentists has issued the following appeal, in which it will be seen that volunteers are asked for the identical purpose proposed by the National Security League. The circular reads as follows:

"The time has come! Our Nation is facing one of the gravest crises in its history! As a profession we are in position to render to our Government and to our fellow men a service not to be equaled by any other organization of specialists. Will we accept the challenge? Positively and unflinchingly, Yes! We will rise to the fullest needs of the emergency and prove our value and sincerity as a public service agency in our important field.

"It is truly said that an army fights on its stomach and teeth. This statement covers the whole situation. As monitors of the teeth we are supervisors of the stomach, hence, an army is helpless without our professional offices. We must, therefore, become a united army within ourselves and throw our whole energy into the work that is before us.

"First of all we must have organization. Next must be formulated sane and sound plans for action. These plans must be carried forward by men of capability, influence and experience. The Preparedness League of American Dentists supplies the necessary organization and the following names represent the directors and dictators of the League: Board of Trustees, Thomas P. Hinman, Harvey J. Burkhart, Otto U. King and Frank W. Low; Advisory Board, representing the National Dental Association, E. C. Kirk, Lafayette L. Barber, Herbert L. Wheeler, H. E. Friesell, and S. D. Boak, Dental Corps, United States Army.

"It is needless to comment on the peculiar fitness of these men nor to refer to the value of their united efforts in forwarding any movement for the welfare of humanity of our great profession. These men love their chosen calling, live for it each day and no effort or sacrifice within their power is too great to make its usefulness more potent.

"We want *you* and every other member of the National Dental Association to join the League and agree, if possible, to prepare the mouth of at least one applicant for enlistment to the National Guard, Army or Navy, who has been rejected because of defective oral conditions and is unable to pay for the necessary dental service, so that he may meet the requirements of the examining officers. We request that all such work be reported to the League headquarters.

"Next we want you, if possible, to join the Officers Reserve Corps, Dental Section, of the Army and equip yourself by joining a Sectional Unit of the League in your city or neighborhood and study the prescribed course in oral and dental surgery which will prepare you to pass the examination for entrance to this corps. Successful applicants are commissioned as First Lieutenants with salary of $2,000 a year when in actual service. There is neither number nor age limit and none will be called to service except in event of actual war. The League will supply application blanks and has the assurance of the Surgeon General's Department that less formality will be required of applicants vouched for by this organization.

"Our ambition is to give our Government the largest, most efficient and most representative Officers Reserve Corps, Dental Section, of any nation of the world. This we owe to our country and to our profession alike. Do *your* part today and thus assure its accomplishment.

"All further information regarding Reserve Corps, Sectional Units, etc., may be obtained at League Headquarters. Those wishing to join the Corps, kindly send full name, whether or not a member of the N. D. A., college and date of graduation. If not already a member of the League, we earnestly request you to become so by enclosing one dollar ($1.00) for active membership. This is much needed to cover postage, printing and other necessary expenses, the clerical work being done gratis by the secretaries of the officers of the League. Address, Preparedness League of American Dentists, 3 Professional Bldg., 131 Allen St., Buffalo, N. Y.''

All loyal American dentists should respond promptly, enlisting with the League for whatever service each may feel able to render.—Editorial in *Items of Interest* for April.

